# Quantitative Proteomic Profiling of Mitochondrial Toxicants in a Human Cardiomyocyte Cell Line

**DOI:** 10.3389/fgene.2020.00719

**Published:** 2020-07-07

**Authors:** Zhengxi Wei, Jinghua Zhao, Jake Niebler, Jian-Jiang Hao, B. Alex Merrick, Menghang Xia

**Affiliations:** ^1^National Center for Advancing Translational Sciences, National Institutes of Health, Bethesda, MD, United States; ^2^Poochon Scientific, Frederick, MD, United States; ^3^Division of the National Toxicology Program, National Institute of Environmental Health Sciences, National Institutes of Health, Durham, NC, United States

**Keywords:** mitochondrial toxicant, FCCP, dinoterb, picoxystrobin, pinacyanol, triclocarban, global proteomic profiling, pathway enrichment analysis

## Abstract

Mitochondria are essential cellular organelles that participate in important cellular processes, including bioenergetics, metabolism, and signaling. Recent functional and proteomic studies have revealed the remarkable complexity of mitochondrial protein organization. Mitochondrial protein machineries with diverse functions such as protein translocation, respiration, metabolite transport, protein quality control and the control of membrane architecture interact with each other in dynamic networks. The goal of this study was to identify protein expression changes in a human cardiomyocyte cell line treated with several mitochondrial toxicants which inhibit mitochondrial membrane potential (MMP) and mitochondrial respiration. AC16 human cardiomyocyte cells were treated with carbonyl cyanide *p*-(trifluoromethoxy)phenylhydrazone (FCCP), dinoterb, picoxystrobin, pinacyanol, and triclocarban for 18 h around the IC_50_ values generated from MMP assay. The samples were harvested and labeled with tandem mass tags with different mass isotopes. Peptide assignment was performed in Proteome Discoverer. Each dataset was analyzed in Ingenuity Pathway Analysis (IPA). In the proteomic profile, these compounds showed dysregulation of a group of mitochondrial proteins (e.g., NDUA, NDUB, BCS1, CYB5B, and SDHF2), as well as proteins involved in lipid metabolism (e.g., CPT, MECR, and LPGAT1), cytoskeleton protein changes (e.g., CROCC, LAMC3, FBLN1, and FMN2) and stress response (e.g., IKBKG, IKBB, SYVN1, SOD2, and CPIN1). Proteomic data from the current study provides key insights into chemical induced cellular pathway dysregulation, supporting the use of proteomic profiling as a sensitive method to further explore molecular functions and disease pathogenesis upon exposure to environmental chemicals.

## Introduction

Mitochondria, the intracellular powerhouse, generate chemical energy in the form of ATP through the process of oxidative phosphorylation (Wallace et al., 1997). The oxidative phosphorylation process requires delivery of hydrogen in the form of NADH and FADH_2_ to complexes I and II, respectively, flux of electrons along complexes II and III to oxygen, delivery of ADP and inorganic phosphate to ATP synthase, and formation of an electrochemical gradient through the movement of protons across the mitochondrial inner membrane.

Many mitochondrial toxicants may impact mitochondrial function either directly or indirectly. Toxicants with direct impact will modulate mitochondrial function by binding directly to specific mitochondrial targets, such as the oxidative phosphorylation complexes I–V or the MPTP, or by interfering with a specific mitochondrial process, including inhibition of mitochondrial DNA replication or modulation of mitochondrial resident enzymes in the TCA cycle and fatty acid metabolism pathways. Toxicants with indirect impacts may modulate other intracellular processes which occur outside the mitochondria that eventually impact mitochondrial function. For example, increase in cellular oxidative stress induce mitochondrial ROS. Imbalances in cytoplasmic Ca^2+^ levels also dissipate MMP.

The mechanisms responsible for mitochondrial toxicity are very diverse, and a single chemical may impact mitochondrial function in multiple ways through distinct processes. The consequences of these adverse effects (either a single effect or a combination of multiple effects) include altered bioenergetics and interference in other key metabolic pathways. In the past decade, OMICS technologies have evolved as important tools in the advancement of personalized medicine. One such technology, MitoChip, has been used as a transcriptomics tool for elucidation of mechanisms associated with mitochondrial toxicity. The mouse MitoChip incorporates 542 oligonucleotides that represent the mitochondrial pathway and biological processes (Will and Dykens, 2018). As more mitochondrial proteins have been identified and annotated (Morgenstern et al., 2017), quantitative proteomic analysis has enabled direct measurement of protein expression changes, which indicate final outcomes of altered signaling and metabolism regulation. Usually, characterization of mitochondrial proteome used mitochondria isolated from tissues. Taylor et al. (2003) reported on mitochondria proteome derived from human heart tissue. They have found that the lysate of purified mitochondria contains cytoplasm proteins which are normally associated with mitochondria. According to the finding from the mitochondrial human proteome project (HPP) initiated by the Italian HPP group (Fasano et al., 2016), mitochondrial proteome should be taken into account as a dynamic and possibly chronosteric reality, with significant overlaps with the whole-cell proteome. Some research groups have used the whole lysate from the AC16 cells. For example, Luo et al. (2014) used AC16 cells as a model in a transcriptome study and the proteomic approach was used to confirm that the regulated transcriptome alters the cellular proteome. In the field of toxicology, the primary goal is to understand how chemicals affect pathways or proteins in a biological context. Yoon et al. (2018) performed proteomics and metabolomics analyses in spinochrome D (SpD)-treated AC16 cells to explain SpD’s cardioprotective effect against doxorubicin toxicity. The purpose of the current study was to evaluate how a mitochondrial toxicant affects cellular proteins, we used the whole lysate of AC16 cells, which provides the context of how cytoplasmic protein/signaling affects mitochondrial proteins in cardiomyocytes. This is the first report of a quantitative proteomics study obtained from mitotoxicant treated human cardiomyocytes. Furthermore, network analysis provides a more comprehensive understanding of how different proteins interact with specific signaling or metabolic pathways, which enables further prediction of potential pathology changes. For example, in doxorubicin treated cardiomyocytes, down regulation of proteins associated with oxidative phosphorylation, fatty acid metabolism, and the TCA cycle impair cardiac energy metabolism and induce apoptotic genes to promote the loss of cardiomyocyte, leading to cardiac hypertrophy (Vijay et al., 2016).

Previously, we performed qHTS of the Tox21 10K compound collection, which consists of drugs as well as environmental and industrial chemicals, by using a MMP assay as an initial screening step in HepG2 cells to identify chemicals that may have adverse effects on mitochondria (Sakamuru et al., 2012; Attene-Ramos et al., 2013, 2015). In the previous study, the primary screening prioritized around 600 chemicals, 35 of which were selected for tier 2 screening using ROS, p53 and Nrf2/ARE assays, and were further evaluated for their mechanism of action in assays including cellular respiration, Parkin translocation, and *C. elegans* ATP status (Xia et al., 2018). From the previous study, we found that MMP inhibitors impair mitochondrial function through different mechanisms of action, which motivated us to further investigate these compounds in a specific biological context. Due to the bioenergetic importance of mitochondria in cardiac cellular functions, we chose human AC16 human cardiomyocytes (Davidson et al., 2005) for further evaluation of selected mitotoxicants. In the current study, we performed a global proteomic analysis of AC16 human cardiomyocyte cells following treatment with a well-known mitochondria uncoupler *p*-triflouromethoxyphenylhydrazone (FCCP), as well as four under-characterized mitochondrial toxicants (dinoterb, picoxystrobin, pinacyanol and triclocarban) in order to quantify global protein level changes caused by these mitochondrial toxicants. Changes in mitochondrial protein expression, along with alterations in non-mitochondrial protein expression were found after treatment with these mitochondrial toxicants. Changes in expression of proteins involved in metabolism and redirection of energy usage were also closely related to mitochondria dysfunction. These findings provide new insights into the comprehensive evaluation of mitochondrial dysfunction caused by the test compounds that were included in this study.

## Materials and Methods

### Cell Culture

The human cardiomyocyte cell line (AC16) was purchased from MilliporeSigma (Burlington, MA, United States). AC16 cells were maintained and grown in DMEM/F12 supplemented with 2 mM EmbryoMax L-glutamine, 12.5% EmbryoMax fetal bovine serum (FBS) and 1% of EmbryoMax Penicillin–Streptomycin Solution at 37°C in a humidified atmosphere of 5% carbon dioxide (CO_2_). Cell culture medium and supplements were purchased from Sigma-Aldrich (St. Louis, MO, United States).

### Mitochondrial Membrane Potential and Cell Viability Assays

The MMP assay (Codex Biosolutions, Montgomery Village, MD, United States), a fluorescence-based assay, was used to quantify changes in MMP following compound treatment. AC16 cells were plated at a density of 1000 cells/well and a volume of 5 μL/well in black collagen-coated 1536-well clear-bottom assay plates. The plates were then incubated for 16 h to allow cell adhesion. After the 16 h incubation period, 23 nL of test compounds ranging from 1.4 nM to 20 mM dissolved in DMSO (final concentrations in the assay were 6.4 pM to 92 μM) were transferred to the assay plates using a Wako Pintool station (Wako Automation, San Diego, CA, United States). The cells were treated for 5 h or 24 h at 37°C, after which 5 μL of Mito-MPS dye loading solution was added to each well. The assay plates were then incubated at 37°C for 30 min before measuring the fluorescence intensity at 490 nm excitation and 535 nm emission for green fluorescent monomers, and 540 nm excitation and 590 nm emission for red fluorescent aggregates using an Envision plate reader (PerkinElmer, Shelton, CT, United States). Data was expressed as the ratio of 590 nm/535 nm emissions values. After measurement of MMP, 5 μL Celltiter-Glo (Promega, Madison, WI, United States) was added to each well and luminescence signal was measured through ViewLux plate reader (Perkin Elmer, Waltham, MA, United States) after 30 min incubation at room temperature. Relative cell viability was measured by relative luminescence units.

### Compound Treatment and Harvesting Cultured Cells for Proteomic Profiling

AC16 human cardiomyocytes (passage 7) were cultured in T-75 cm^2^ flasks at densities of 2 × 10^6^. After incubation at 37°C for 6 h, the media was replaced with fresh culture medium containing test compounds or DMSO for 18 h treatment. The cells were harvested by scraping off tissue culture plates after washing twice with PBS. The homogenates were centrifuged at 200 × *g* for 5 min at 4°C. The cell pellets were stored at −80°C for proteomic profiling.

### Protein Extraction and Trypsin Digestion

The cell pellets were lysed in 0.4 ml lysis buffer (20 mM Tris-HCl, pH 7.5, 150 mM NaCl, and 2% SDS) followed by 10 times of a 10 s pulse sonication with 10 s rest between each time (Fisher Scientific Sonic Dismembrator Model 500, 15% amplitude). The lysate was centrifuged at 15,000 × *g* for 10 min at 4°C. Supernatants were collected and stored at −80°C for further analysis. The protein concentration of the supernatants was determined by a BCA^TM^ Reducing Reagent compatible assay kit (Thermo Scientific, Rockford, IL, United States). For trypsin digestion, an aliquot of 150 μg proteins were reduced with DTT followed by alkylation with iodoacetamide for 30 min at RT in the dark. Alkylated proteins were desalted on Amicon Ultra Centrifuge Filter Units (EMD Millipore) followed by trypsin digestion at 37°C overnight (16 h). After trypsin digestion, the samples were dried and stored at −80°C.

### Tandem Mass Tag (TMT) Labeling

The TMT-11plex labeling method tags 11 samples with different mass isotopes in one batch. However, triplicates of each treatment group were prepared (18 cell lysate samples total), which exceeded the number of samples that could be tagged in a single set. To test all 18 cell lysate samples, we divided them into two sets. The first set included nine samples (triplicates of DMSO, FCCP and dinoterb) and two pooled samples. The second set also included nine samples (triplicates from pinacyanol, picoxystrobin, and triclosarban) and two pooled samples. Pooled samples were used as internal standards in both sets, which were created by mixing all 18 digested peptide samples equally. Tryptic digested peptides and the reference peptides were labeled with 11plex TMT reagents according to the manufacturer’s instructions (Thermo Scientific). Peptides labeled by different TMT reagents were then mixed and dried using a Speed-Vac.

### Peptide Fractionation by Basic Reverse-Phase Liquid Chromatography

Extensive fractionation by basic reverse-phase liquid chromatography was used to reduce sample complexity and thus reduce the likelihood of peptides being co-isolated and co-fragmented. The 11plex TMT labeled sample was resuspended in 20 μL of 10 mM TEAB (triethylammonium bicarbonate) buffer before being separated on an Agilent 1290 Infinity UHPLC system mounted with a Poroshell HPH-C18 Column (2.1 × 150 mm, 2.7 μm, Agilent). The labeled peptides were eluted using a gradient of 2–100% Solvent B (10 mM TEAB, pH 8.0, 90% ACN) at a flow rate of 200 μL/min over 120 min. A total of 96 fractions were collected in a 96 well plate, which were then concatenated to 24 fractions by combining every four sequential elutes, vacuum dried and stored at −80°C until further LC/MS-MS analysis.

### Nanospray LC/MS-MS Analysis and Database Search

LC/MS-MS analysis was carried out using a Thermo Scientific Q-Exactive hybrid Quadrupole-Orbitrap Mass Spectrometer and a Thermo Dionex UltiMate 3000 RSLCnano System. Each peptide fraction was loaded into a peptide trap cartridge at a flow rate of 5 μL/min. The trapped peptides were eluted onto a reversed-phase 20 cm C18 PicoFrit column (New Objective, Woburn, MA, United States) using a linear gradient of ACN (3–36%) in 0.1% formic acid. The elution duration was 100 min at a flow rate of 0.3 μL/min. Eluted peptides from the PicoFrit column were ionized and sprayed into the mass spectrometer using a Nanospray Flex Ion Source ES071 (Thermo Scientific) under the following settings: spray voltage, 1.8 kV, capillary temperature, 250°C.

The Q Exactive instrument was operated in the data dependent mode to automatically switch between full scan MS and MS/MS acquisition. Survey full scan MS spectra (m/z 350-1800) were acquired in the Orbitrap with 35,000 resolution (m/z 200) after an accumulation of ions to a 3 × 10^6^ target value based on predictive AGC. The maximum injection time was set to 100 ms. The 15 most intense multiply charged ions (*z* ≥ 2) were sequentially isolated and fragmented in the octopole collision cell by higher-energy collisional dissociation (HCD) using normalized HCD collision energy 30 with an AGC target 1 × 10^5^ and a maximum injection time of 400 ms at 17,500 resolution. The isolation window was set to 2. The dynamic exclusion was set to 20 s. Charge state screening was enabled to reject unassigned and 1+ and 7+ or higher charge states ions.

### MS Peptide Assignment

MS Raw data files (24 files from 24 fractions) were searched against the Human UniProtKB human protein sequence databases (20,608 entries, download on December 10, 2016) and mitochondrial protein annotations were obtained from the NCBI website using the Proteome Discoverer 1.4 software (Version 1.4.1.14, Thermo Fisher, San Jose, CA, United States) based on the SEQUEST and Percolator algorithms (Spivak et al., 2009). The searches were performed with the following parameters: The false positive discovery rates (FDR) was set to 5%. The resulting Proteome Discoverer Report contained all assembled proteins with peptides sequences and peptide spectrum match counts (PSM#) and TMT-tag based quantification ratios. Relative protein abundance was calculated as the ratio of sample abundance to reference abundance using the summed reporter ion intensities.

### Statistical Analysis

Student’s *t*-test was used to analyze the statistical significance of differences between DMSO controls and compound treated groups. The average, standard deviation, coefficient of variation (CV) and *T*-test (*p*-values) were calculated using Microsoft Excel. Power value calculations were completed using TTestIndPower. power in R (cutoff: α = 0.05, β = 0.2). The correlation analysis was performed using CORREL function in Microsoft Excel. The annotation of GO molecular function and biological process categories were analyzed using the UniprotKB protein database online tool. The datasets from all five different drug treatments were uploaded to Ingenuity Pathway Analysis (Qiagen, Redwood City, CA, United States) for pathway enrichment analysis. Further pathway annotation was obtained from publicly available bioinformatics resources, such as GO and KEGG, Panther (PANTHER version 14), and the Reactome Pathway Database^1^.

### IPA Analysis

The datasets of five treatment groups were uploaded to IPA software (version: 51963813) for core analysis. The cutoff filters were set to: power value > 0.8 and ratio of treatment group over DMSO greater than 1.15 or less than 0.8. The top five enriched pathways ranked by *p*-value from IPA were reported.

## Results

### Selection of Working Concentrations of Mitochondrial Toxicants

**FIGURE 1:**
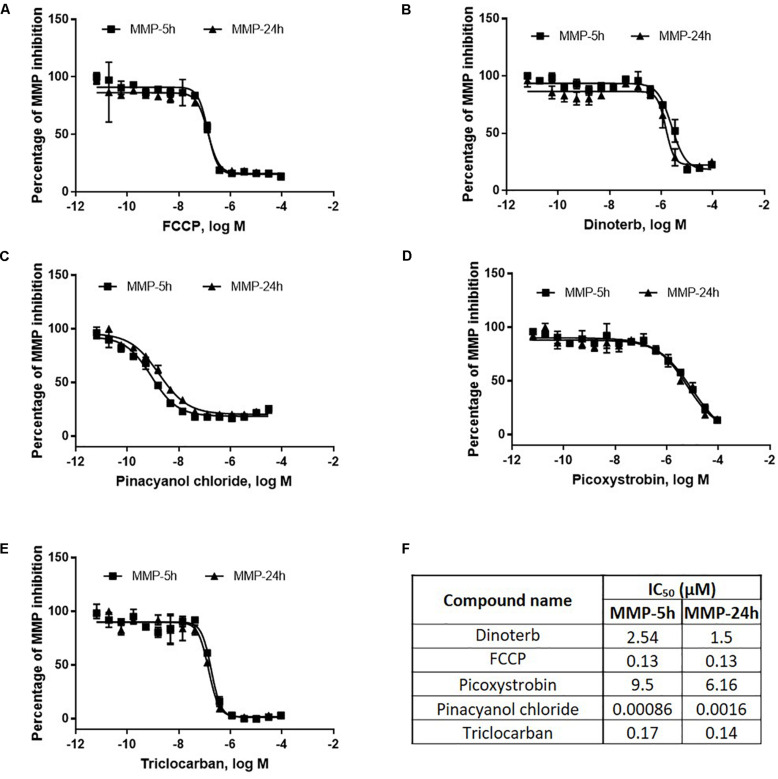
Concentration-response of mitochondria toxicants, FCCP **(A)**, dinoterb **(B)**, pinacyanol chloride **(C)**, picoxystrobin **(D)**, and triclocarban **(E)** in MMP assay. IC_50_ values **(F)** of MMP and cytotoxicity assays were listed for these five compounds. Inactive compound was defined by its efficacy of less than 20% compared to DMSO control. AC16 cells were treated by each compound in multiple concentrations ranging from 6.4 pM to 92 μM for 5 h, or 24 h. IC_50_ – IC_70_ values of compounds that decreased MMP at 5 h were used as treatment condition in proteomics study.

To select the optimal treatment concentrations of mitochondrial toxicants for the proteomic study, AC16 cells were treated with FCCP, dinoterb, picoxystrobin, pinacyanol, triclocarban in the MMP assay (Sakamuru et al., 2012). All of these compounds decreased MMP in a concentration-dependent manner after 5 or 24 h treatment (Figure 1). As shown in Figures 1A–E, the inhibitory effects of FCCP, dinoterb, pinacyanol, picoxystrobin, and triclocarban in MMP were unchanged with prolonged treatment. IC_50_ values of MMP inhibition and cytotoxicity for these compounds are listed in Figure 1F. The IC_50_ values to induce cytotoxicity were 10-fold higher than the IC_50_ values to inhibit MMP. Therefore, concentrations around IC_50_ values of MMP inhibition, which were non-cytotoxic, were chosen as working concentrations for the proteomics study.

### Proteomic Study Process and Data Quality

**FIGURE 2:**
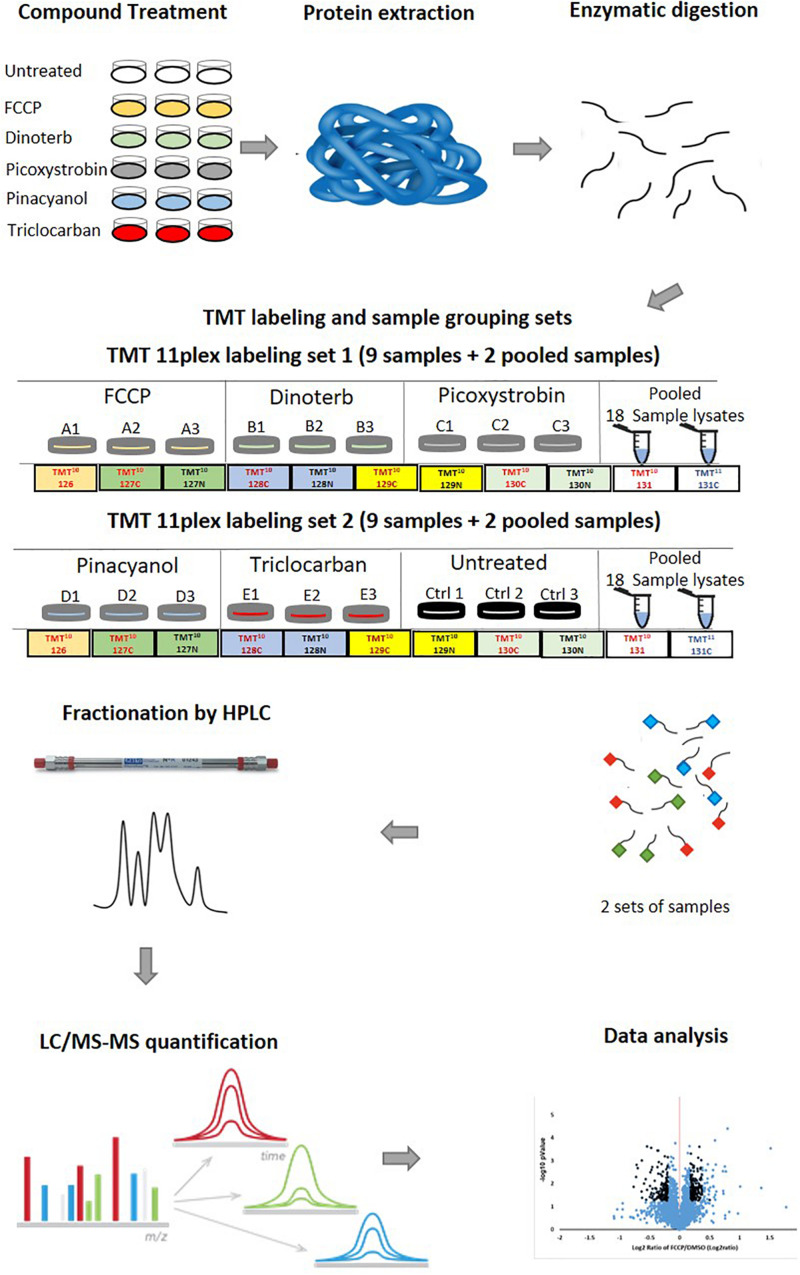
A standardized mass spectrometry-based quantitative proteomic profiling workflow. The workflow includes the preparation of cell lysate, trypsin digestion, TMT 11-plex labeling of tryptic peptides, fractionation of labeled peptides by reverse-phase UHPLC, LC-MS/MS analysis, database search, quantification analysis and data analysis. Firstly, AC16 cells were treated with FCCP, dinoterb, pinacyanol, picoxystrobin and triclocarban at a single concentration around the identified IC_50_ value. Each compound was tested in biological triplicates. Secondly, proteins from all 18 samples were extracted and digested after 18 h treatment. To accommodate the TMT-11plex labeling method, 18 samples were divided into two sets. Set 1 included samples treated with FCCP, dinoterb, and picoxystrobin. Set 2 included samples treated with pinacyanol, triclocarban, and untreated DMSO group. Each set contained two pooled samples containing all 18 individual samples as an internal control. Thirdly, the labeled peptides were separated by UHPLC and analyzed in LC-MS/MS. Lastly, the identified peptide was quantified and the proteomic dataset was analyzed in IPA.

We performed global proteomic analyses on proteins from AC16 cells treated with five mitochondrial toxicants or DMSO (vehicle control). This study was developed in order to obtain robust and interpretable data that can be used to develop an overview of protein level dysregulation due to stress and disruption of mitochondrial homeostasis. The process is described schematically in Figure 2, including sample treatment, preparation, LC/MS-MS analysis, and data analysis which were described in the “Materials and Methods” section. The proteomic dataset was uploaded to PRIDE (accession number: PXD019076).

A total of 6,024 proteins were detected of which 4,309 proteins (Supplementary Table S1) overlapped between 5 treatment groups and DMSO control on peptide analysis and assignment. This group of overlapping proteins was established as the user dataset for IPA analysis and in the treatment-specific pathway enrichment analysis. Since the 18 samples were divided into two sets (Figure 2), the CV of each detected protein in pooled samples from both sets represents reproducibility of the proteomics study. The CV ratios of the 4,309 proteins from pooled samples in sets 1 and 2 are plotted in Supplementary Figure S1A. A majority of the protein expression have small variations with CV < 5%. The symmetrical distribution of CV ratio values of four pooled samples in set 1 and 2 indicated the analysis of two sets of samples were reproducibly processed and analyzed. The CV distribution of samples from individual treatment group and mixed samples (four pooled samples) were similar (Supplementary Figure S1B).

Among the 4,309 proteins, proteins with significant changes in expression from each treated sample were filtered out by applying a threshold. For pathway analysis, the significant change of each individual protein affects the related pathway calculation. *P*-value is used to determine statistical significance. Power value is the probability that the statistical hypothesis test detects the difference in the sample if that difference really exists. In this study, each sample has only three replicates and the effect size is relatively small. Therefore, we used a student *t*-test power value as the cutoff (α = 0.05, β = 0.2) in the pathway analysis to minimize type I and type II error. Supplementary Figure S2A indicates the number of significantly changed proteins (power > 0.8) in each compound treatment group. Each treatment has significantly up/down regulated around 10% of the 4,309 proteins. This study investigated samples from acutely treated cells, so that large protein fold-changes in protein expression were not observed. Since, there was low variability in samples generated from the same batch of cells, we used 0.15-fold of change of protein expression as the cutoff. Supplementary Figure S2B indicates the number of significantly changed (power > 0.8) proteins when applying the threshold of more than 0.15-fold change. When applying 0.15-fold of change as the additional cutoff criterial, FCCP, dinoterb and picoxystrobin treatment modulated a larger number of protein changes than pinacyanol and triclocarban.

### Overview of Pathway Enrichment From Ingenuity Pathway Analysis

To better understand how these mitochondrial toxicants affect homeostasis of cardiomyocytes, proteomic datasets of each chemical treatment were analyzed and compared using IPA software. The thresholds used in IPA to identify proteins with significant changes in expression were a power value greater than 0.8 and an expression change greater than ±0.15-fold. Furthermore, not all proteins could be detected in our analysis due to the detection limit of MS. To exclude any detection bias, we performed pathway enrichment analyses using both the IPA default database and the user dataset (4,309 commonly detected proteins in all 18 samples) as a reference set. As shown in Table 1, the enriched pathways were much more specific to molecule’s function level when using the user dataset as a reference. Some overlap in significantly enriched pathways that were found, which is expected regardless of the reference set that was used for analysis (overlapping pathways are indicated in bold font).

**TABLE 1:** Pathway enrichment analysis by Ingenuity Pathway Analysis.

	IPA default dataset enriched pathway (*p*-value)	User data set enriched pathway (*p*-value)
FCCP	• Epithelial adherens junction signaling (1.16E-03) • ILK signaling (1.46E-03) • **Acute phase response signaling (2.83E-03)** • Germ cell-Sertoli cell junction signaling (3.72E-03) • **4-1BB signaling in T lymphocytes (4.47E-03)**	• **Acute phase response signaling (1.70E-02)** • iNOS signaling (3.00E-02) • Hepatic fibrosis/hepatic stellate cell activation (4.27E-02) • **4-1BB signaling in T lymphocytes (4.41E-02)** • Cell cycle regulation by BTG family proteins (4.41E-02)
Dinoterb	• **CDP-diacylglycerol biosynthesis I (7.45E-03)** • **Phosphatidylglycerol biosynthesis II (non-plastidic) (8.66E-03)** • Glutathione biosynthesis (1.55E-02) • Hypusine biosynthesis (1.55E-02) • **Cell cycle regulation by BTG family proteins** (1.59E-02)	• **CDP-diacylglycerol biosynthesis I (1.82E-02)** • **Phosphatidylglycerol biosynthesis II (non-plastidic) (1.82E-02)** • Lactose degradation III (2.70E-02) • Triacylglycerol biosynthesis (2.82E-02) • **Cell cycle regulation by BTG family proteins (6.03E-02)**
Picoxystrobin	• Sirtuin signaling pathway (9.35E-05) • **Cell cycle regulation by BTG family proteins (1.79E-04)** • EIF2 signaling (5.71E-04) • **D-MYO-INOSITOL-5-PHOSPHATE METABOLISM (5.84E-04)** • Iron homeostasis signaling pathway (8.48E-04)	• **Cell cycle regulation by BTG family proteins (3.60E-03)** • Estrogen-mediated S-phase entry (1.15E-02) • **D-MYO-INOSITOL-5-PHOSPHATE METABOLISM (1.52E-02)** • Nicotine degradation III (1.58E-02) • Melatonin degradation I (1.58E-02)
Pinacyanol	• **ILK signaling (2.01E-04)** • **Oleate biosynthesis II (animals) (5.18E-04)** • **Semaphorin signaling in neurons (5.28E-04)** • **Granzyme B signaling (7.94E-04)** • **Acute phase response signaling (1.26E-03)**	• **Oleate biosynthesis II (animals) (1.77E-03)** • **ILK signaling (4.09E-03)** • **Acute phase response signaling (4.46E-03)** • **Semaphorin signaling in neurons (6.80E-03)** • **Granzyme B signaling (7.63E-03)**
Triclocarban	• **Chemokine signaling (1.28E-03)** • Germ cell-Sertoli cell junction signaling (1.60E-03) • Regulation of actin-based motility by rho (2.04E-03) • **Adenine and adenosine salvage VI (2.67E-03)** • **PAK signaling (3.35E-03)**	• **Chemokine signaling (9.39E-03)** • *S*-Methyl-5′-thioadenosine degradation II (1.38E-02) • **Adenine and adenosine salvage VI (1.38E-02)** • Lactose degradation III (1.38E-02) • **PAK signaling (1.97E-02)**

*Bold font indicates the overlapping enriched pathways in IPA default dataset and user data set.*

The overview of pathway enrichment analysis across these different compound treatments suggest that the most significantly enriched pathways can be grouped based on biological process. (1) Metabolism: carbohydrate metabolism such as lactose degradation III (triclocarban); Lipid metabolism such as CDP-diacylglycerol biosynthesis (dinoterb) and oleate biosynthesis II (pinacyanol). Amino acid metabolism such as hypusine biosynthesis (dinoterb); Nucleotide metabolism such as adenine and adenosine salvage (triclocarban), sirtuin signaling pathway (picoxystrobin). (2) Cytoskeleton: such as ILK signaling (pinacyanol), PAK signaling (triclocarban), epithelial adherens junction signaling (FCCP, triclocarban), (3) Stress-induced events such as acute phase response (FCCP, pinacyanol), cell cycle regulation by BTG family proteins (dinoterb, picoxystrobin).

### Treatment-Specific Analysis of Dysregulated Pathways From Combined Datasets

**FIGURE 3:**
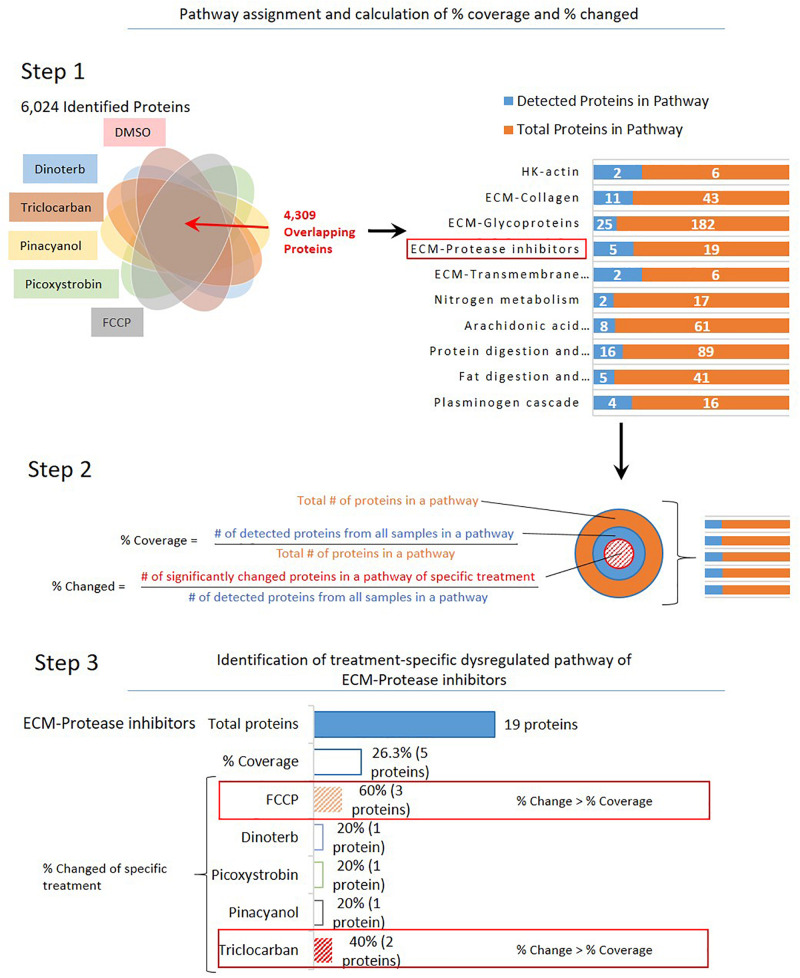
Overview of selection for dysregulated pathways in treatment-specific analysis. Step 1: A total of 4,309 overlapped proteins in all six groups of samples were assigned to 186 pathways based on annotation. Step 2: Calculation of percent of coverage and percent of dysregulation. Step 3: An example (ECM-protease inhibitors) to show selection criteria of specific treatment: Percent of dysregulation in a single treatment group is greater than percent coverage of all samples.

**FIGURE 4:**
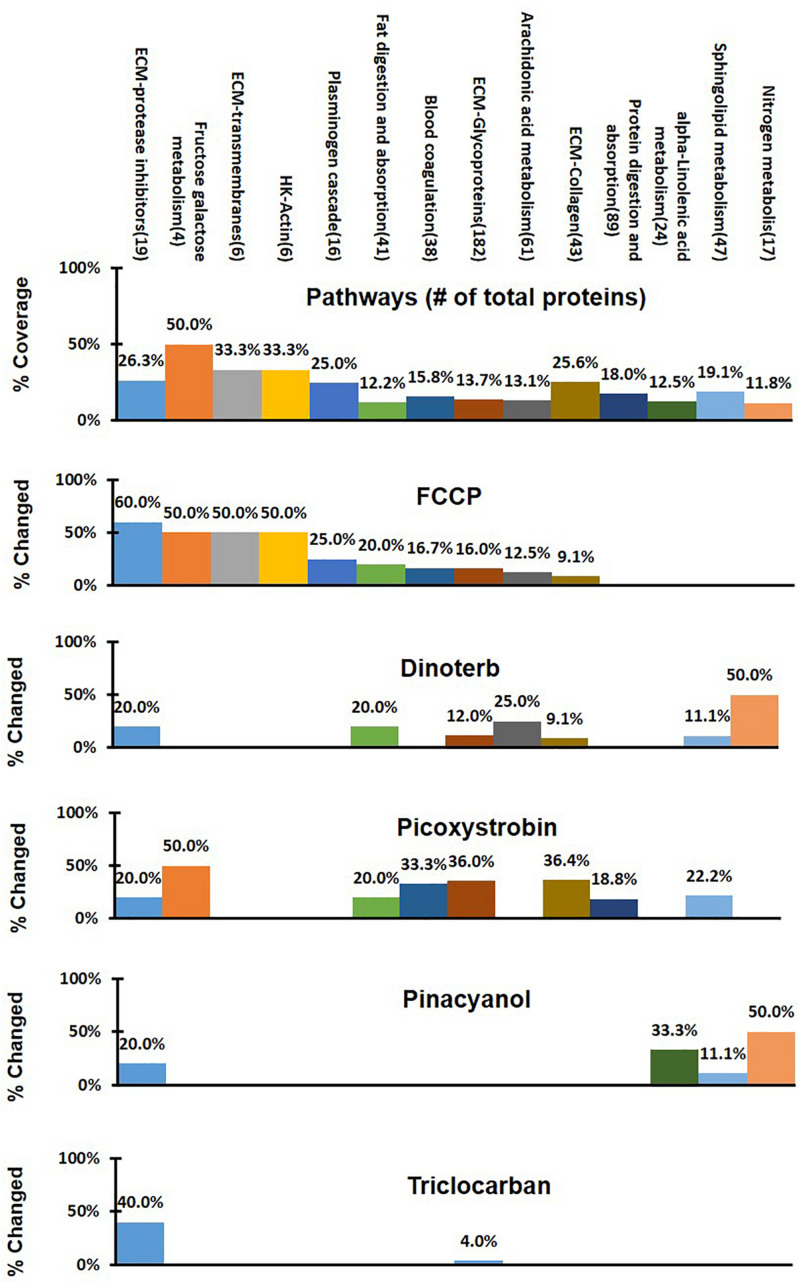
Comparison of different treatment effects on the 14 selected pathways in treatment-specific analysis. Percent of detected proteins in each pathway was shown in the first panel as percent of coverage from all treatment groups. Percent of changed protein in a specific pathway upon different treatments was shown under the percent of coverage, respectively. The colored bars in the five lower panels indicated treatment-specific dysregulated pathway.

To identify pathways that may have shown significant treatment-specific dysregulation, a 3-step analysis process was used, as shown in Figure 3. A treatment-specific dysregulated pathway in this analysis is defined as distinguished up- or down-regulated pathway upon a compound treatment when compared to other treatments. Firstly, the 4,309 proteins that were commonly detected across DMSO control and five compound treatment groups were assigned into 186 pathways according to annotations from the public domain (Supplementary Table S2). Secondly, percent coverage of each pathway was calculated by dividing the number of commonly detected proteins in that pathway in all treatment samples over the total number of known proteins that are annotated in the pathway. Percent change of each pathway was defined as the percentage of significantly changed proteins in each treatment group, which was calculated by dividing the number of significantly changed proteins (power cutoff > 0.8) over the total number of proteins that are detected in that pathway across all samples. Lastly, the treatment-specific pathways were found if the percent change of a pathway was higher than or equal to the percent coverage. In Figure 4, we listed 14 pathways which exhibited treatment-specific dysregulation. All five treated groups altered protein expression in the ECM-protease inhibitor pathway, but FCCP and triclocarban treatment had higher or equal percent changed than percent coverage in the ECM-protease inhibitor pathway, indicating that this pathway showed treatment-specific dysregulation in the FCCP and triclocarban treatment groups (shown as an example in Figure 3, step 3). Picoxystrobin mostly changed proteins in ECM-glycoprotein and ECM-collagen pathways. FCCP, dinoterb, and picoxystrobin significantly enriched proteins annotated with the functions of fat digestion and absorption. FCCP and picoxystrobin also affected fructose-galactose metabolism. Dinoterb and pinacyanol both affected nitrogen metabolism. Dinoterb significantly affected arachidonic acid metabolism. Generally, besides ECM and cytoskeleton related pathways and targets revealed in IPA analysis, the treatment-specific analysis based on GO/KEGG annotations provides additional information regarding impairment of metabolism.

### Analysis of Significantly Changed Mitochondrial Proteins

**FIGURE 5:**
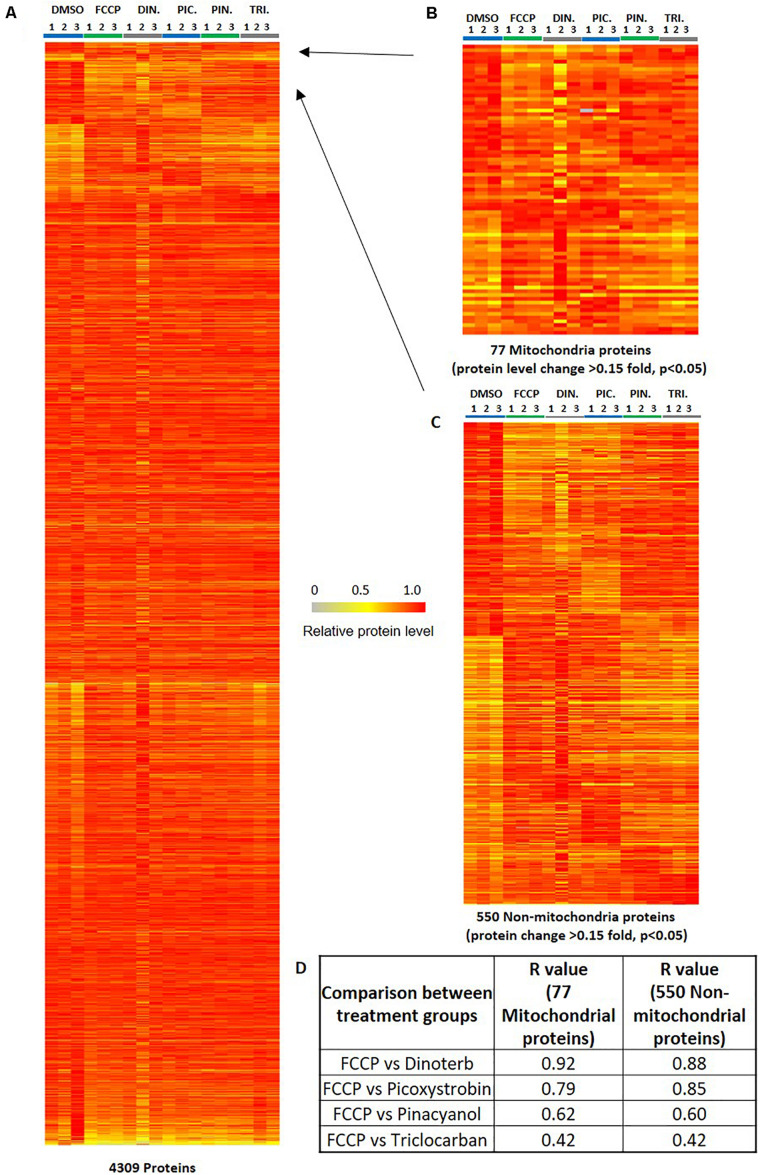
Heat map of illustrating protein expression level. **(A)** The distribution of 4,309 commonly detected proteins in all samples (triplicates in six groups). **(B)** The differential expression of ranked 77 mitochondrial proteins. The ranked proteins were significantly (*p* < 0.05, *n* = 3) up/down-regulated 0.15-fold in at least one compound treatment across six groups of 18 samples. **(C)** The differential expression of ranked 550 non-mitochondrial proteins. The ranked proteins were significantly (*p* < 0.05, *n* = 3) up/down-regulated 0.15-fold in at least one compound treatment across six groups of 18 samples. The color key indicates the relative expression level of each protein (0 to 1.0) across 18 samples. The relative protein level of the sample with highest expression level in all 18 samples was defined as 1. **(D)** The analysis for the correlation of relative protein level of the 77 mitochondrial proteins or 550 non-mitochondrial proteins between positive control FCCP and other compound treatment groups. The calculation of *R*-value is performed using the excel correlation function based the ratio value of compound treatment/DMSO.

**FIGURE 6:**
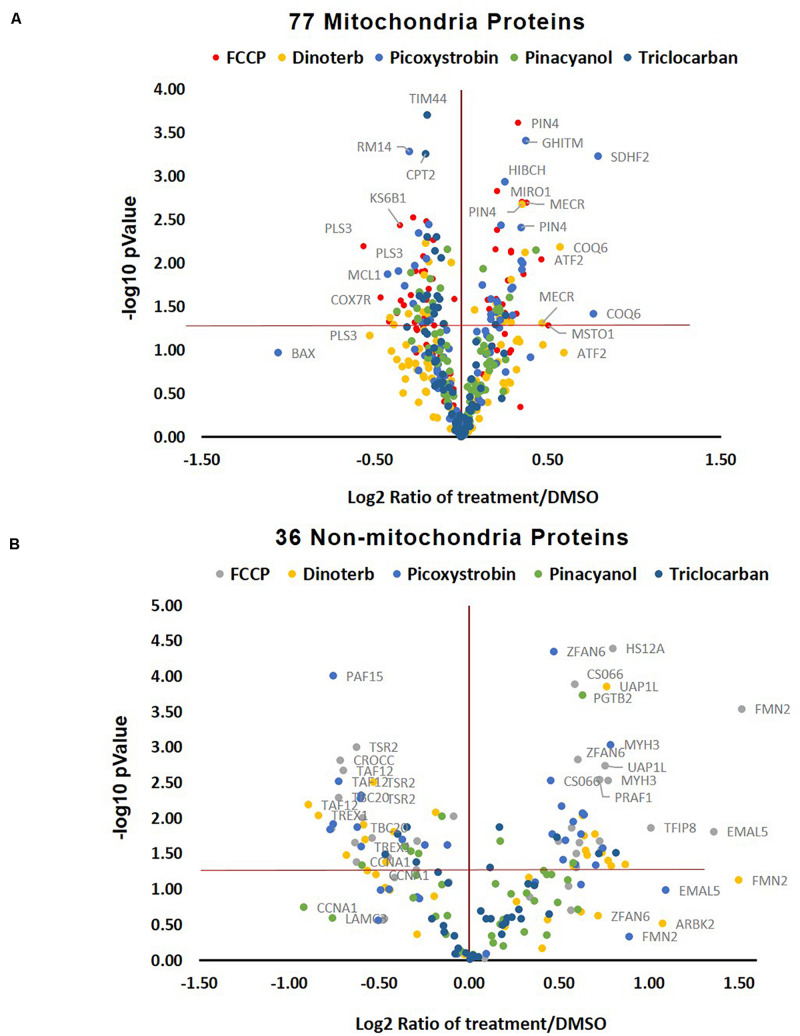
Volcano plot of changed protein expression and *p*-value upon different treatments. **(A)** The distribution of ranked 77 mitochondrial proteins. The ranked proteins were significantly (*p* < 0.05, *n* = 3) up/down-regulated 0.15-fold in at least one compound treatment across six groups of 18 samples. **(B)** The distribution of top ranked 36 non-mitochondrial proteins. The ranked proteins were significantly (*p* < 0.05, *n* = 3) up/down-regulated 0.5-fold in at least one compound treatment across six groups of 18 samples.

**FIGURE 7:**
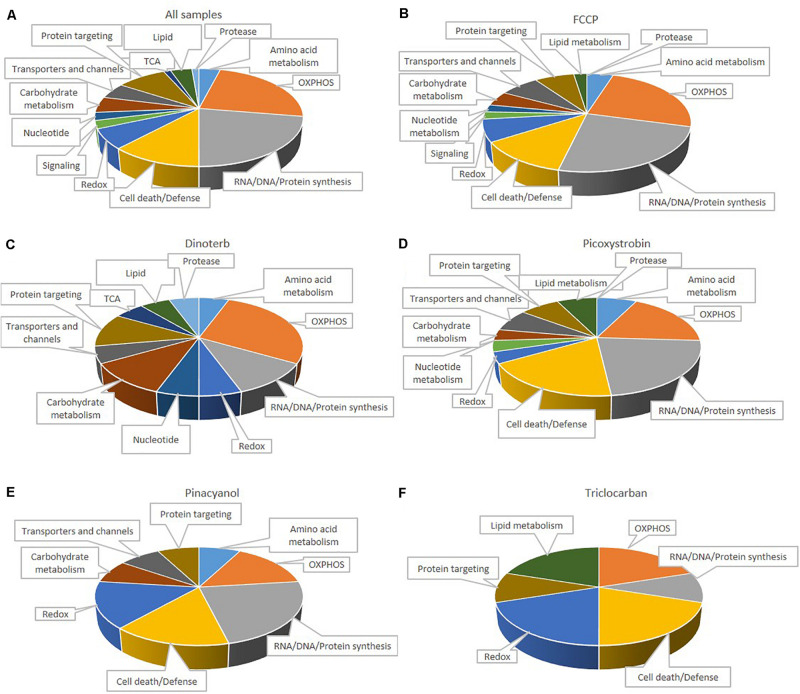
Distribution of 77 functionally classified mitochondria proteins in all samples **(A)**, FCCP **(B)**, dinoterb **(C)**, picoxystrobin **(D)**, pinacyanol **(E)** and triclocarban **(F)**. Assignments were made on the basis of classification of Taylor et al. (2003), KEGG, and information provided in the NCBI nucleotide database.

Many mitochondrial pathways have been thoroughly studied and to a large extent, many proteins within these pathways are known. According to the annotations of cellular protein localization from UniProt, 502 mitochondrial proteins were commonly detected in all samples. 77 mitochondrial proteins (Supplementary Table S3) were significantly (*p* < 0.05) up- or down-regulated more than 0.15-fold in at least one chemical treatment. The heat map in Figure 5B showed the relative protein level of these 77 proteins across the five treatment groups, as well as the DMSO negative control. FCCP, dinoterb, picoxystrobin, pinacyanol and triclocarban affected expression of 41, 18, 27, 13, and 10 mitochondrial proteins, respectively. FCCP and dinoterb were most correlated when compared the top-ranked mitochondrial in correlation analysis (*R* = 0.92) (Figure 5D). As shown in Figure 6A, among the proteins with significant changes in expression (−log10 *p*-value > 1.3, above the horizontal red line), mimitin (MIMIT, NDUF2), MECR, peptidyl-prolyl *cis–trans* isomerase NIMA-interacting 4 (PIN4), and FRDA/FXN were upregulated across three of the treatment groups. CYB5B and AUHM were downregulated in three of the treatment groups. The functions of these significantly changed proteins across multiple treatment groups, cover the oxidation-reduction process, negative regulation of insulin secretion involved in cellular response to glucose stimulus, the fatty acid biosynthetic process, rRNA processing, heme biosynthesis, and the branched-chain amino acid catabolic processes. The perturbation of mitochondrial proteins clearly indicated the disruption of homeostasis in energy metabolism. As summarized in Figure 7, these 77 mitochondrial proteins were sub-grouped based on function into oxidative phosphorylation (OXPHOS), cell death/defense, redox, lipid metabolism, nucleotide metabolism, carbohydrate metabolism, TCA cycle, RNA/DNA/Protein synthesis, transport, cell death/defense, signaling, protease, and protein targeting functions as reported in previous studies on the human heart mitochondrial proteome (Taylor et al., 2003). Proteins involved in OXPHOS, RNA/DNA/protein synthesis, protein targeting and redox were generally dysregulated across all five treatment groups.

### Analysis of Significantly Changed Non-mitochondrial Proteins

Five hundred and fifty non-mitochondrial proteins were significantly (*p* < 0.05) up- or down-regulated more than 0.15-fold in at least one chemical treatment (Figure 5C and Supplementary Table S4). The distribution of the top ranked 36 proteins in volcano plot indicated that FCCP and picoxystrobin affected most of top-ranked non-mitochondria proteins among five treatment groups (Figure 6B). The 36 top-ranked proteins mainly function in maintaining structure, cell cycle regulation, metabolism, transcriptional regulation and DNA replication and repair. For example, ECM related proteins such as LAMC3, FBLN1, and ITIH3 were significantly down-regulated in FCCP or picoxystrobin treated groups. CCNA1 and CROCC are cell cycle progression related proteins and were down-regulated in FCCP and dinoterb treatment groups. Also, dinoterb and picoxystrobin are more correlated with FCCP when compared the top-ranked non-mitochondrial proteins in correlation analysis (*R* = 0.88 and 0.85). UAP1L which is involved in carbohydrate metabolism was upregulated in FCCP, dinoterb, and picoxystrobin groups. Transcription initiation factor TFIID subunit 12 (TAF12) which mediates regulation of RNA polymerase transcription was downregulated in FCCP, dinoterb and picoxystrobin groups. FMN2, TREX1, PAF15, and protein arginine *N*-methyltransferase 6 (ANM6) are involved in cellular response to DNA damage, and were dysregulated in FCCP and picoxystrobin treatment groups.

## Discussion

After characterization of mitochondria proteins, further research focused on the applications of mitochondrial dysfunction, such as cardiotoxicity (Petricoin et al., 2004; Hoffmann et al., 2012), and neurotoxicity (Li et al., 2012; Schultz et al., 2015). AC16 cells, a human cardiomyocyte cell line, have been used as a model for proteomics and metabolomics in cardiotoxicity study. To explore the effects of mitochondria toxicants in AC16 cells, the global proteomic profiles of the vehicle control (DMSO), FCCP, dinoterb, picoxystrobin, pinacyanol and triclocarban treated groups were compared. A total of 4,309 proteins (Supplementary Table S1 and Figure 5A) were reproducibly detected across all these samples with Proteome Discoverer software. According to the annotations, we further analyzed the enriched pathways in IPA, as well as pathways based on analysis of publicly available annotations for treatment-specific dysregulation. Certain differentially expressed proteins (Supplementary Table S5) which may explain the chemical’s toxicity character based on functional and biological annotations such as mitochondrial, non-mitochondrial, metabolism, cytoskeleton, and stress-induced events are discussed in the following sections. This study serves as a proof of concept for proteomic profiling proteins or pathways related to mitochondria dysfunction. The biological validations of these altered proteins or pathways are being planned for future study.

### Pathway Analysis

Within the IPA analysis, pathways that were enriched in the treatment groups were typically divided into several categories – pathways related to stress response, cytoskeleton, and metabolism-related pathways. From IPA pathway enrichment analysis, we found some proteins that contributed to the enrichment of multiple pathways. These proteins were listed since they are connecting nodes for different enriched pathways. In the FCCP treatment group, pathways involved in acute phase response and 4-1BB signaling were enriched. The overlapping proteins in these pathways include an increase of inhibitor of nuclear factor kappa B kinase subunit gamma (IKBKG, NEMO) and NFKB inhibitor beta (NFKBIB, IKBB) which are closely related to inhibition of the NFKB complex and further discussed in the stress-induced events section. In the dinoterb treatment group, enriched pathways included CDP-diacylglycerol biosynthesis I, phosphatidylglycerol biosynthesis II (non-plastidic) and cell cycle regulation by BTG family proteins. The commonly down-regulated proteins in CDP-diacylglycerol biosynthesis I and phosphatidylglycerol biosynthesis II (non-plastidic) pathways are AGPAT1 and LPGAT1. AGPAT1 and LPGAT1 regulate triacylglycerol biosynthesis which is critical to keep phospholipid balance and maintain mitochondria membrane structure integrity. The dysregulation of these enzymes involved in triacylglycerol biosynthesis were further discussed in section “Proteins Involved in Lipid Metabolism”. The picoxystrobin treatment group affected cell cycle regulation through changes in expression of BTG family proteins and D-myo-inositol-5-phosphate metabolism pathways. A commonly up-regulated protein in cell cycle regulation by BTG family proteins within the dinoterb and picoxystrobin treatment groups was CNOT7. Pathway enrichment analysis of the pinacyanol treatment group was nearly identical no matter which reference was used as background. The common upregulated protein in acute phase response, semaphore signaling in neurons and ILK signaling is mitogen-activated protein kinase 3 (MAPK3, MK03). MAPK3, which is also known as ERK1/2, is widely involved in cell signaling pathways for proliferation, migration, growth, differentiation, phosphorylation, apoptosis, homeostasis, DNA damage response, and survival. It is expected that MAPK3 is likely the connecting node of the 3 pathways. The overlapping enriched pathways in the triclocarban treatment group are mainly cell adhesion and cytoskeleton related signaling. The representative proteins are decreased ITGA4 and elevated CFL1, which are further discussed in the cytoskeleton section.

IPA pathway enrichment analysis considered each treatment group as an individual dataset in identifying pathways that were significantly enriched within that group. However, IPA analysis cannot compare the treatment groups side by side on a specific pathway. Therefore, we compared the percent change of protein in 186 pathways among all treatment groups. When percent change of protein in a pathway upon a treatment is larger than percent coverage of detected protein in that pathway, the treatment dysregulated that pathway. Fourteen pathways were distinguished in treatment-specific analysis, which were listed in Figure 4. The commonly changed proteins which contributed to dysregulated pathways are given here. FCCP and picoxystrobin affected fructose-galactose metabolism pathway through GALE. Dinoterb and pinacyanol both affected nitrogen metabolism pathway through GLNA, which is a glutamate-ammonia ligase.

### Mitochondria Related Proteins

IPA analysis (default background) results showed that FCCP, picoxystrobin and pinacyanol have enriched pathways which are related to mitochondrial dysfunction. Common proteins among these pathways include NDUFA and CY1. OXPHOS was the most affected category in mitochondria proteins (Figure 7). FCCP decreased the expression of multiple components of the OXPHOS pathway, including NADH dehydrogenase (NDUA4 and NDUB5, referred to subunits of Complex I), CY1, COX7R (also referred to as Complex IV), and ATP8 (also referred to as Complex V). These results indicated that more detailed analysis of OXPHOS is necessary to better understand the dysregulation of mitochondria function. We found that the expression level of 28% of annotated proteins associated with the OXPHOS pathway (11 out of 39) were inhibited when data from all five compound treatment groups were compiled together.

LC/MS-MS accurately detected the changed expression level of Complex I. The decrease in expression level of different subunits in Complex I suggests it was generally inhibited by FCCP, dinoterb, picoxystrobin and triclocarban. Dinoterb, picoxystrobin and triclocarban also inhibited NADH dehydrogenase (NDUA4, NDUB5), and the inhibition of Complex I was previously observed in rat liver mitochondria treated with picoxystrobin and pinacyanol (Xia et al., 2018). Our proteomic findings are consistent with previous observations that FCCP was previously characterized as a Complex I and V inhibitor (Li et al., 2006; Pravdic et al., 2012). Complex I (NADH dehydrogenase) utilizes NADH generated from glycolysis and the TCA cycle to pump protons out of the mitochondrial matrix. It is the largest enzyme complex in the electron transport chain, containing 45 subunits. The subunits are assembled together in a coordinated manner and acting intrinsically or transiently for constructing Complex I (Lazarou et al., 2009; Andrews et al., 2013). The deficiency of Complex I reportedly has a pathogenesis role in rare and common human disease (DiMauro and Schon, 2003; Schapira, 2008; Mimaki et al., 2012).

Complex II is responsible for the oxidation of succinate to fumarate, which delivers additional electrons to coenzyme Q through FAD. Once Complex I is inhibited, an increase in expression of SDHAF2 may be a cellular response to increased activity of Complex II and maintains functioning in the OXPHOS pathway due to the role of Complex II as another protein responsible for electron donation to coenzyme Q. We observed that succinate dehydrogenase assembly factor 2 (SDHAF2, SDHF2), which is involved in the formation of succinate dehydrogenase (Complex II), as well as the covalent attachment of FAD to the SDHA subunit of Complex II (Hao et al., 2009), was upregulated in the picoxystrobin and pinacyanol treatment groups. However, SDHA and SDHB (two of four subunits of Complex II) were detected in the proteomic study and did not show changes in expression across any of the treatment groups. Mitochondrial chaperone BCS1, which is primarily involved in assembly of Complex III, was also significantly downregulated in the FCCP and dinoterb treatment groups.

Another important cause of MRC inhibition is the impairment of the TCA cycle (Fernández-Vizarra et al., 2009; Kühl et al., 2017). We speculated the following imbalance of biochemical reactions may lead to MRC inhibition. FCCP and dinoterb down-regulated IDH3B, which plays a structural role in the assembly and function of isocitrate dehydrogenase for catalyzing the decarboxylation of isocitrate into alpha-ketoglutarate. The reduction of isocitrate dehydrogenase activity results in less NADH production, which is needed to drive MRC. Picoxystrobin and pinacyanol up-regulated SDHAF2. Succinate dehydrogenase is also involved in the TCA cycle, which results in the production of FADH_2_ that can subsequently be used by additional succinate dehydrogenase within the electron transport chain to facilitate ATP production in response to decreases in NADH dehydrogenase expression and NADH synthesis. Fourteen kDa phosphohistidine phosphatase (PHP14) was also downregulated in four of the five treatment groups. PHP14 is involved in the negative regulation of ATP citrate synthase, which is the enzyme in the TCA cycle that converts oxaloacetate back into citrate. Decreased activity of ATP citrate synthase from increased expression of PHP14 can decrease TCA cycle activity, and subsequently result in cell damage (Krieglstein et al., 2008). In this discovery phase, the dysregulation of complexes I and II, as well as TCA cycle enzymes are prioritized to be studied for mitochondria toxicity.

### Proteins Involved in Lipid Metabolism

Based on the information from pathways enrichment analysis, the overall significantly up/down-regulated proteins (*p* < 0.05, expression change > ± 0.15 fold) related to metabolism are listed in the Table 2.

**TABLE 2:** Lipid metabolism related proteins.

Gene symbol	Short name	Cellular function	Biological process	FCCP	Dinoterb	Picoxystrobin	Pinacyanol	Triclocarban
CPT2	Carnitine *O*-palmitoyltransferase 2, mitochondrial	Carnitine shuttle	Fatty acid degradation			Down		Down
FADS1	Fatty acid desaturase 1	Alpha-linolenic acid metabolic process	Biosynthesis of unsaturated fatty acids					Down
MECR	Trans-2-enoyl-CoA reductase, mitochondrial	Fatty acid biosynthetic process	Fatty acid elongation	Up	Up	Up		
DHAK	Bifunctional ATP-dependent dihydroxyacetone kinase/FAD-AMP lyase (cyclizing)	Carbohydrate metabolic process	Glycerolipid metabolism			Down		
LCLT1	Lysocardiolipin acyltransferase 1	Cardiolipin acyl-chain remodeling	Glycerolipid metabolism					Down
ACOD	Acyl-CoA desaturase	Fatty acid biosynthetic process	Biosynthesis of unsaturated fatty acids	Down	Down	Down	Down	
FADS2	Fatty acid desaturase 2	Alpha-linolenic acid metabolic process	Alpha-linolenic acid metabolism				Down	
CP51A	Lanosterol 14-alpha demethylase	Cholesterol biosynthetic process	Steroid biosynthesis			Up		
DHCR7	7-Dehydrocholesterol reductase	Blood vessel development	Steroid biosynthesis			Up		
SGPL1	Sphingosine-1-phosphate lyase 1	Androgen metabolic process	Sphingolipid metabolism			Up		
PLCA	1-Acyl-sn-glycerol-3-phosphate acyltransferase alpha	CDP-diacylglycerol biosynthetic process	Glycerolipid metabolism	Down	Down	Down		
LGAT1	Acyl-CoA: lysophosphatidylglycerol acyltransferase 1	Glycerophospholipid biosynthetic process	Glycerophospholipid metabolism	Down	Down	Down		
SPTC2	Serine palmitoyltransferase 2	Ceramide biosynthetic process	Sphingolipid metabolism			Down		
CBR3	Carbonyl reductase [NADPH] 3	Cognition	Arachidonic acid metabolism		Up			
NSMA3	Sphingomyelin phosphodiesterase 4	Cellular response to tumor necrosis factor	Sphingolipid metabolism		Down			
GPX1	Glutathione peroxidase 1	Aging	Arachidonic acid metabolism	Down	Down			
DHC24	Delta(24)-sterol reductase	Amyloid precursor protein catabolic process	Steroid biosynthesis	Down				

Lipid metabolism was dysregulated in all treated samples. The enzymes of fatty acid oxidation in animal cells are localized to the mitochondrial matrix. Several important enzymes were discussed by their functions such as (1) fatty acid metabolism and (2) glycerolipid metabolism. Mitochondria is the major organelle for fatty acid β-oxidation (Fromenty and Pessayre, 1995; Houten et al., 2016). Whereas short-chain fatty acid and medium-chain freely enter the mitochondria, the entry of long-chain fatty acid into the mitochondria depends on carnitine (Roberts et al., 2015). Several enzymes function in the carnitine shuttle, including carnitine palmitoyltransferases I and II which are anchored on the outer and inner mitochondrial membranes, respectively, and function together in the transport of long-chain fatty acids into the mitochondria for fatty acid β-oxidation. Picoxystrobin and triclocarban both decreased expression of CPT2, which converts acyl-carnitine esters back to acyl-coenzyme A esters within the mitochondria. Downregulation of this protein leads to the accumulation of long-chain fatty acids in the cytosol and decrease in carnitine. A correlation exists between decrease of plasma concentrations of carnitine and mitochondrial dysfunction (Adeva-Andany et al., 2017). Inhibition of fatty acid oxidation is one of the mechanisms of mitochondrial toxicity. Therefore, the inhibition of CPT2 in the picoxystrobin and triclocarban treatment groups confirmed their mitochondrial toxicity through this mechanism of action. Interestingly, enoyl-[acyl-carrier-protein] reductase (MECR), an enzyme which functions in fatty acid elongation, was upregulated in FCCP, dinoterb and picoxystrobin treated samples. The upregulation of fatty acid elongation is reciprocal to the inhibition of fatty acid degradation as the effect of inhibition CPT2. FCCP, dinoterb and triclocarban regulated triacylglycerol biosynthesis through down-regulating AGPAT1, LPGAT1, and LCLT1, respectively. These enzymes are critical to catalyze the reacylation of lysophosphatidylglycerol to phosphatidylglycerol, a membrane phospholipid that is an important precursor for the synthesis of cardiolipin. Cardiolipin is an essential phospholipid of the inner mitochondrial membrane. It is the only known dimeric phospholipid, and its unique structure plays a major role in maintaining the function of membrane-associated proteins in the mitochondria (Horvath and Daum, 2013). The direct effects on cardiolipin can acutely undermine mitochondrial function. The decreased expression levels of AGPAT1, LPGAT1 and LCLT1 in these samples suggest the potential of dampening mitochondria membrane structure.

Overall, it’s clear that reduction of energy production from the mitochondria affects carbohydrate, lipid, and amino acid metabolism. Changes in anabolic pathways may result in impairment of mitochondrial structure integrity.

### Proteins Involved in Cytoskeleton

IPA pathway analysis enrichment indicated that FCCP, pinacyanol, and triclocarban affected pathways related to cellular morphology. The common proteins of the enriched pathways from different chemical treatments were RAS related kinases and cytoskeleton related structural proteins. It’s known that cytoskeletal network is important for mitochondrial arrangement and regulation in muscle cells. We observed that FCCP decreased tubulin and actin related proteins (CROCC and FMN2) in this study as reported in skeletal muscle cells (Kuruvilla et al., 2003). FCCP, picoxystrobin, and triclocarban decreased expression of laminin (LAMC3) and fibulin (FBLN1), which are an important component of the extracellular matrix. Increased CFL1 in the triclocarban treatment group indicated actin depolymerization. Also, the decrease in ITGA4 suggested less cell adhesion with the ECM. The decrease of actin stability, microtubule formation and extracellular matrix proteins indicate the disassembly of cytoskeleton. In contrast with FCCP or triclocarban, pinacyanol increased FBLIM1 which localizes at cell junctions and functions as a force-activated mechanosensor, suggesting increased cell adhesion.

### Proteins Involved in Stress-Induced Events

The disruption of MMP disturbs ATP synthesis due to the loss of the electrochemical gradient across the mitochondrial membrane. MRC inhibition induces a significant ATP shortage, which leads to cell death. All five compounds increased apoptosis related mitochondrial proteins. There are so many factors that could trigger cell death including cellular stress, DNA damage, and ROS generation. It is interesting that the upregulation of apoptosis related proteins were mostly located among proteins that are localized to the mitochondria. For example, FCCP and picoxystrobin increased ATF2 which is involved in anti-apoptosis and DNA damage response. FCCP and dinoterb also upregulated GCLC which is an enzyme involved in glutathione biosynthesis and maintains cell redox homeostasis. Previous studies indicated FCCP and dinoterb were the most potent compounds in inducing ROS and Nrf2-ARE activities among the five treatment groups that were included in this study (Xia et al., 2018). The upregulation of GCLC may be a response to oxidative stress caused by Complex I inhibition. FCCP and pinacyanol also showed increases in expression of NFKB essential modulator (IKBKG, NEMO) and NFKB inhibitor beta (NFKBIB, IKBB), which is a component of the IKK complex and is involved in phosphorylating IkB prior to ubiquitination and degradation. However, FCCP also increased expression of NF-kappa-B inhibitor beta (IKBB), which is responsible for binding to NFkB and keeping it in its inactive state, which decreases the anti-apoptotic activity of NFkB. FCCP, dinoterb, picoxystrobin upregulated E3 ubiquitin-protein ligase synoviolin (SYVN1) which mainly protects cells from ER stress-induced apoptosis. Pinacyanol increased endonuclease G (NUCG) which is a protein related to DNA fragmentation induced-apoptosis. The down-regulation of SOD2, which functions to clear mitochondria generated ROS, in the pinacyanol treatment group indicated the loss of buffering capability of oxidative stress. Triclocarban increased both pro- and anti-apoptotic protein expression, including BAX and CPIN1. This indicates that the cells may undergo early apoptosis to counter-balance cell survival signaling.

## Conclusion

FCCP, dinoterb, picoxystrobin, pinacyanol, and triclocarban were reported as mitochondrial toxicants (Xia et al., 2018) and were further investigated for their effects on cellular proteins and pathways using a proteomic approach. The proteomic profiling data from this study suggested that the dysregulated proteins in mitochondria and metabolism fit the general mechanisms of mitochondrial dysfunction. (1) FCCP, dinoterb, picoxystrobin, and triclocarban decrease the expression of Complex I, but only FCCP inhibited Complex III, IV and ATP synthase (Complex V) expression. (2) Picoxystrobin and triclocarban inhibited fatty acid β-oxidation through down-regulating CPT2 (Wallace and Starkov, 2000; Dykens and Will, 2007). The pathway enrichment analysis further revealed that these compounds affect lesser-known pathways or proteins which are related to mitochondrial toxicity. For example, FCCP, dinoterb and triclocarban inhibited the biosynthesis of cardiolipin precursor through down-regulating AGPAT1, LPGAT1, and LCLT1. The dysregulation of triacylglycerol biosynthesis may result in structural impairment. The general adverse effects such as collapse of cytoskeleton, stress-induced events were also observed across all 5 compounds treatment. Our results characterize the differences of proteins in a cardiomyocyte cell line treated with mitochondrial toxicants, and indicate that the dysregulation of glycolytic, gluconeogenic and anabolic enzymes and mitochondrial proteins may lead to changes in OXPHOS, lipid, carbohydrate, amino acid, and nucleotide metabolism.

## Data Availability Statement

Our mass spectrometry proteomics data have been deposited to the ProteomeXchange Consortium via the PRIDE partner repository with the dataset identifier PXD019076.

## Author Contributions

MX designed study. JZ and J-JH executed the experiments. ZW and J-JH performed data analysis. ZW and JN wrote the manuscript. BM and MX edited the manuscript. All authors contributed to the article and approved the submitted version.

## Conflict of Interest

J-JH was employed by Poochon Scientific, Frederick, MD, United States. The remaining authors declare that the research was conducted in the absence of any commercial or financial relationships that could be construed as a potential conflict of interest.

## Footnotes

1 https://reactome.org/PathwayBrowser/

## Supplementary Material

FIGURE S1 Characterization of quantity of proteomic profiling results. **(A)** Correlation of coefficient of variation in set 1 and set 2 samples. **(B)** Coefficient of variation distribution of six different groups and a mixture of all 18 samples.

FIGURE S2 Overview of up/down-regulated protein in different treatment groups. **(A)** The number of significantly changed proteins when applying the cutoff of power > 0.8. **(B)** The number of significantly changed proteins when applying the cutoff of power > 0.8 and threshold ± 0.15-fold.

TABLE S1 Compiled information for protein identifications. Accession number, statistical information, sequence coverage and protein annotation information are included.

TABLE S2 Pathway annotation in Poochon database and calculation of coverage percentage.

TABLE S3 The list of 77 non-mitochondrial proteins which were significantly (*p* < 0.05, *n* = 3) up/down-regulated 0.15-fold in at least one compound treatment across six groups of 18 samples.

TABLE S4 The list of 550 non-mitochondrial proteins which were significantly (*p* < 0.05, *n* = 3) up/down-regulated 0.15-fold in at least one compound treatment across six groups of 18 samples.

TABLE S5 The expression level of proteins mentioned in the text in different treatment groups.

## Abbreviations

ACN: acetonitrile
AGC: automatic gain control
AGPAT1: 1-acylglycerol-3-phosphate *O*-acyltransferase 1
ANM6: protein arginine-*N*-methyltransferase 6
ATF2: cyclic AMP-dependent transcription factor ATF-2
ATP8: ATP synthase protein
AUHM: methylglutaconyl-CoA hydratase
BCS1: mitochondrial chaperone BCS1
CCNA1: cyclin-A1
CFL1: cofilin 1
CNOT7: CCR4-NOT transcription complex subunit 7
COX7R: cytochrome c oxidase
CPIN1: anamorsin
CPT2: carnitine palmitoyltransferase II
CROCC: rootletin
CY1: cytochrome C1
CYB5B: cytochrome β 5 type B
DTT: dithiothreitol
FBLN1: fibulin-1
FBLIM1: filamin binding LIM protein
FCCP: carbonyl cyanide *p*-(trifluoromethoxy)phenylhydrazone
FMN2: formin-2
FRDA/FXN: frataxin
GALE: UDP-galactose-4-epimerase
GCLC: glutamylcysteine synthetase gamma
GLNA: glutamine synthetase
GO: Gene Ontology
HCD: high-energy collisional dissociation
IDH3B: isocitrate dehydrogenase 3 beta
IKBB: NFKB inhibitor beta
IKBKG: inhibitor of nuclear factor kappa B kinase subunit gamma
IPA: Ingenuity Pathway Analysis
ITGA4: integrin subunit alpha 4
ITIH3: inter-alpha-trypsin inhibitor heavy chain H3
KEGG: Kyoto Encyclopedia of Genes and Genomes
LAMC3: laminin subunit gamma-3
LCLT1: lysocardiolipin acyltransferase 1
LPGAT1: lysophosphatidylglycerol acyltransferase 1
MAPK3: mitogen-activated protein kinase 3
MECR: *trans*-2-enoyl-CoA reductase
MIMIT: mimitin
MK03: mitogen-activated protein kinase 3
MMP: mitochondrial membrane potential
MPTP: mitochondrial permeability transition pore
MRC: mitochondria respiration chain
NDUA4: NADH dehydrogenase 1 alpha subcomplex subunit 4
NDUB5: NADH dehydrogenase 1 beta subcomplex subunit
NDUF2: NADH dehydrogenase 1 alpha subcomplex assembly factor 2
NDUFA: NADH ubiquinone oxidoreductase complex assembly factor
NEMO: inhibitor of nuclear factor kappa B kinase subunit gamma
NFKBIB: NFKB inhibitor beta
NUCG: endonuclease G
OXPHOS: oxidative phosphorylation
PAF15: PCNA-associated factor
PHP14: fourteen kDa phosphohistidine phosphatase
PIN4: peptidyl-prolyl *cis*-*trans* isomerase NIMA-interacting 4
qHTS: quantitative high-throughput screening
ROS: reactive oxygen species
SDHA: succinate dehydrogenase complex flavoprotein subunit A
SDHAF2: succinate dehydrogenase assembly factor 2
SDHB: succinate dehydrogenase complex flavoprotein subunit B
SDHF2: succinate dehydrogenase assembly factor 2
SOD2: superoxide dismutase 2
SYVN1: synoviolin 1
TCA cycle: tricarboxylic acid cycle
TMT: tandem mass tag
TREX1: three-prime repair exonuclease 1
UAP1L: UDP-*N*-acetylhexosamine pyrophosphorylase-like protein.
