# Supplementary figures and images for: Quantitative Proteomic Profiling of Mitochondrial Toxicants in a Human Cardiomyocyte Cell Line
Source: Front Genet. 2020 Jul 7;11:719. doi: 10.3389/fgene.2020.00719 (PMC7358379; doi:10.3389/fgene.2020.00719)

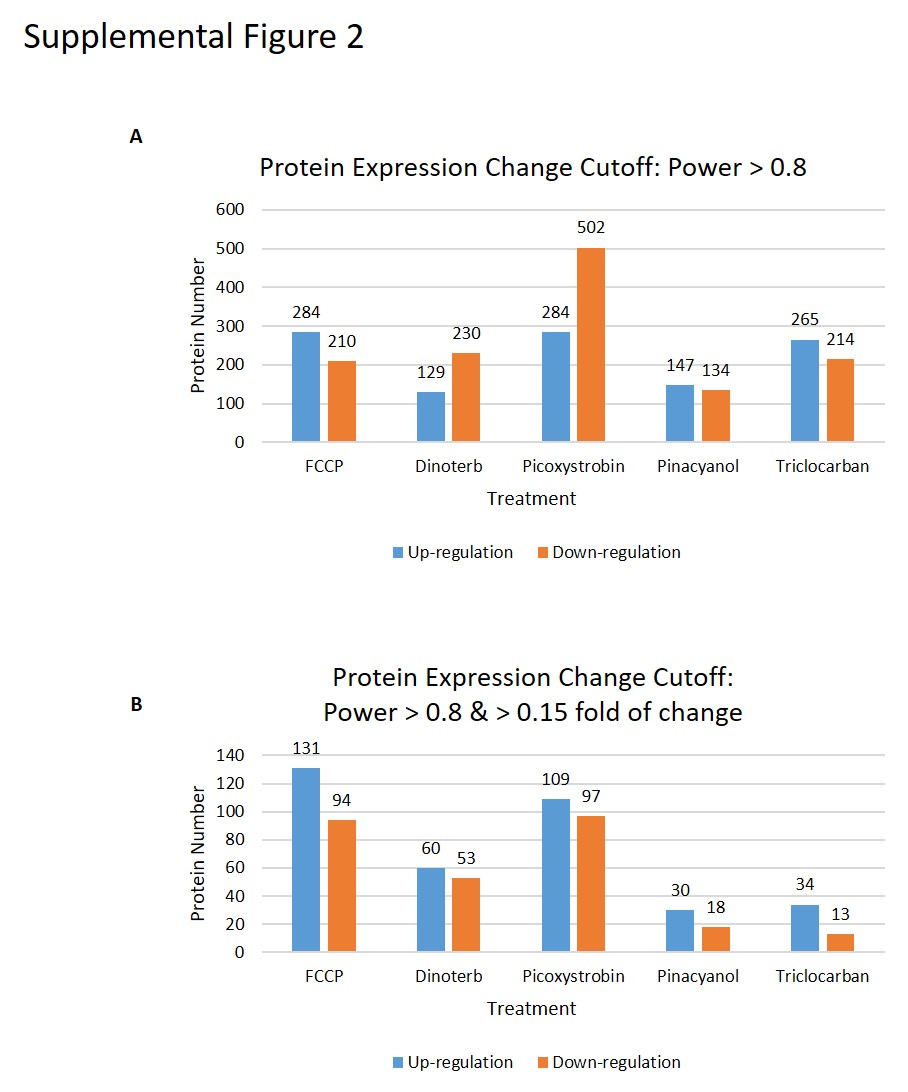

Supplement: FIGURE S1 — Characterization of quantity of proteomic profiling results. (A) Correlation of coefficient of variation in set 1 and set 2 samples. (B) Coefficient of variation distribution of six different groups and a mixture of all 18 samples. [file Image_1.JPEG]

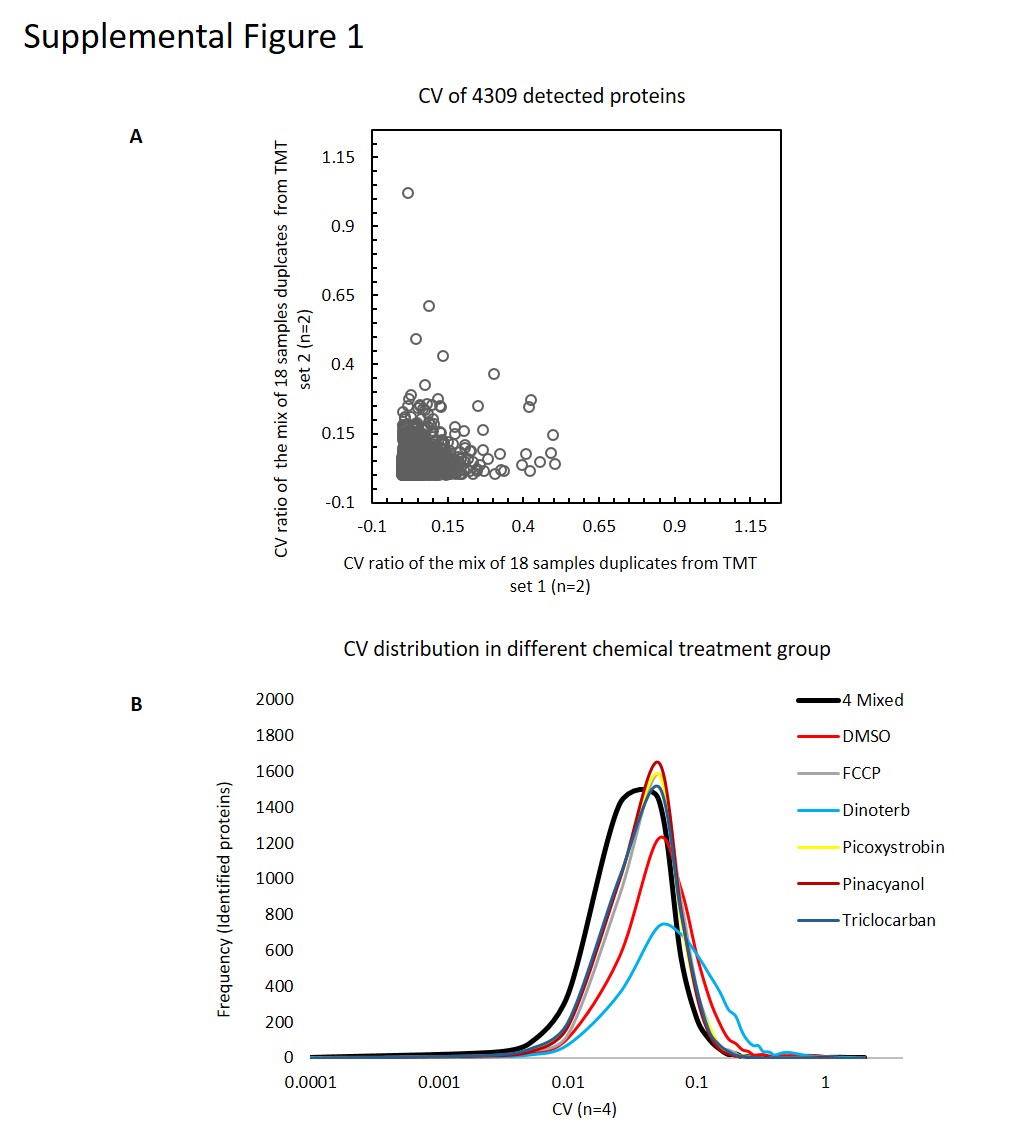

Supplement: FIGURE S2 — Overview of up/down-regulated protein in different treatment groups. (A) The number of significantly changed proteins when applying the cutoff of power > 0.8. (B) The number of significantly changed proteins when applying the cutoff of power > 0.8 and threshold ± 0.15-fold. [file Image_2.JPEG]
